# Financial incentive policies at workplace cafeterias for preventing obesity—a systematic review and meta-analysis (Protocol)

**DOI:** 10.1186/2046-4053-3-128

**Published:** 2014-10-28

**Authors:** Kimi Sawada, Erika Ota, Sadequa Shahrook, Rintaro Mori

**Affiliations:** 1 Department of Nutrition, Faculty of Health Care, Kiryu University, 606-7 Azami, Kasakake, Midori-shi, Gunma Pref 379-2392, Japan; 2 Department of Health Policy, National Center for Child Health and Development, Tokyo, Japan

**Keywords:** Incentive-based, Worksite, Food environmental interventions, Systematic review, Meta-analysis

## Abstract

**Background:**

Various studies are currently investigating ways to prevent lifestyle-related diseases and obesity among workers through interventions using incentive strategies, including price discounts for low-fat snacks and sugar-free beverages at workplace cafeterias or vending machines, and the provision of a free salad bar in cafeterias. Rather than assessing individual or group interventions, we will focus on the effectiveness of nutrition education programs at the population level, which primarily incorporate financial incentive strategies to prevent obesity. This paper describes the protocol of a systematic review that will examine the effectiveness of financial incentive programs at company cafeterias in improving dietary habits, nutrient intake, and obesity prevention.

**Methods/design:**

We will conduct searches in the Cochrane Central Register of Controlled Trials (CENTRAL), MEDLINE, Embase, and PsycINFO.

Interventions will be assessed using data from randomized control trials (RCTs) and cluster RCTs. However, if few such trials exist, we will include quasi-RCTs. We will exclude controlled before-and-after studies and crossover RCTs. We will assess food-based interventions that include financial incentive strategies (discount strategies or social marketing) for workplace cafeterias, vending machines, and kiosks. Two authors will independently review studies for inclusion and will resolve differences by discussion and, if required, through consultation with a third author. We will assess the risk of bias of included studies according to the Cochrane Collaboration’s “risk of bias” tool.

**Discussion:**

The purpose of this paper is to outline the study protocol for a systematic review and meta-analysis that will investigate the effectiveness of population-level, incentive-focused interventions at the workplace cafeteria that aim to promote and prevent obesity. This review will give an important overview of the available evidence about the effectiveness of incentive-based environmental interventions to improve obesity prevention in the workplace and will guide future research in nutrition education and health promotion globally.

**Systematic review registration:**

PROSPERO CRD42014010561

## Background

The proportion of adults who are obese is dramatically increasing worldwide [1,2]. The United States has seen the highest proportion of obesity (body mass index (BMI) ≧30) at 36.5%, while obesity figures have also increased in various developed countries including New Zealand (28.4%), England (24.8%), and Australia (28.3%) [3]. Metabolic syndrome—a collection of conditions that occur together and are associated with obesity including diabetes, high blood pressure, and high cholesterol—is also on the rise.

Obesity is most common in people aged 40–60 years [4]. This age group also represents the largest proportion of workers. The workplace therefore provides a suitable setting to not only deliver dietary interventions to workers of all ages but also to target this high-risk demographic. As people spend more than a third of their day at work, and may access the workplace cafeteria up to 1–2 times or more per day, the cafeteria is an ideal setting to promote behavioral change, particularly targeted at dietary and buying habits [5]. Previous nutrition educational interventions have mostly been carried out at the group or individual level. However, a population approach has a much wider reach and has a greater possibility to change dietary behavior as well as prevent obesity in those who are not yet at risk.

Using a behavioral science theoretical approach that incorporates psychology, sociology, health education pedagogy, and nutrition education is known to be effective in promoting behavioral change. In recent years, efforts focusing on product taxation and pricing strategies have attracted much attention. For example, higher taxes on tobacco products have led to a significant reduction in smoking in the United States, Mexico, and Australia [6-8]. Applying a similar taxation strategy to foods high in fat and sugar has been a hotly debated topic among governments and food and nutrition experts in recent years. Several European countries are currently leading the way in trialing a diverse range of food taxation strategies [9-11], including Hungary [12], which in 2011 introduced a food tax known as the “potato chips tax” to snack foods high in salt and sugar. Various studies are currently investigating ways to prevent lifestyle-related diseases and obesity among workers through interventions at the workplace such as healthy cafeteria menus, point-of-purchase (POP) advertisements [13-16], free meal or discount tickets for healthy meals, price discounts for low-fat snacks and sugar-free beverages at the cafeteria and vending machines, and access to a free salad bar [17-20].

Rather than assessing individual or group interventions, we plan to systematically review the effectiveness of nutrition education programs at the population level that primarily incorporate financial incentive strategies to prevent obesity at a global level.

## Methods/design

### Objectives

To examine the effectiveness of financial incentive policies at workplace cafeterias in improving the dietary habits, nutrient intake, and BMI of employees.

### Type of studies

The review will include randomized control trials (RCTs) and cluster RCTs. If there are few such trials, we will include quasi-RCTs. We will exclude controlled before-and-after studies and crossover RCTs.

This protocol is registered with PROSPERO (International prospective register of systematic reviews) at the National Institute for Health Research and Centre for Reviews and Dissemination (CRD) at the University of York (registration number: CRD42014010561).

### Type of participants

Participants under 65 years of age who are employed at any worksite.

### Type of interventions

We will assess food-based interventions that focus on incentive strategies (discount strategies or social marketing) for workplace cafeterias, vending machines, and kiosks. We will consider calorie counts for food consumption as a co-intervention. If we identify studies that include calorie counts, we will conduct a subgroup analysis with and without calorie counts.

### Type of outcome measures

#### ***Primary outcomes (continuous variables)***

1. Changes in weight [kg]

2. BMI [kg/m^2^]

3. Changes in HbA1c [%]

#### ***Secondary outcomes***

1. Blood pressure [mmHg]

2. Changes in cholesterol levels [g]

3. Food consumption

• changes in vegetable consumption [g or Sv]

• changes in fruit consumption [g or Sv]

• changes in fruit and vegetable consumption [g or Sv]

• changes in consumption of sugary beverages [g or Sv]

• changes in consumption of sweets [g or Sv]

• other foods [g or Sv]

4. Nutritional intake

• changes in fat and oil intake [g]

• changes in fiber intake [mmHg]

• changes in energy intake [kcal]

### Timing of outcomes

•Short-term outcome: Less than 12 months

•Long-term outcome: 12 months or more

### Minimal clinically important difference (MCID)

MCID is defined as more than 5% of body weight.

### Electronic searches

This systematic review will be reported in accordance with the PRISMA statement [21].

We will search the following databases:

1. CENTRAL: monthly searches

2. MEDLINE: weekly searches

3. Embase: weekly searches

4. PsycINFO: weekly searches

We will include all languages in our searches. Our search strategy will be reviewed by an experienced librarian from the National Center for Child Health and Development. An additional file shows the search strategy in more detail [see Additional file 1].

### Searching other resources

#### ***Language: all languages***

We will aim to identify additional studies by hand-searching journals and conference proceedings; searching reference lists of relevant studies, included studies, systematic reviews, and meta-analyses; and reviewing the titles and abstracts of these studies.

### Data collection and analysis

#### ***Selection: inclusion criteria***

1. Participants: employees at any worksite. We will include both genders.

2. Study design: RCTs (including cluster RCTs).

3. Intervention site: cafeterias, vending machines, and kiosks at the worksite.

4. Intervention: organizational-based, food-based incentive pricing strategies and social marketing.

#### ***Selection: exclusion criteria***

1. Excluded studies: observational studies, quasi-experimental designs, and crossover designs.

2. Excluded intervention programs:

• Fitness- and exercise-focused interventions

• Programs that do not use behavioral science theoretical approaches incorporating incentive-pricing strategies for food and social marketing

• Only individualized education programs

3. Excluded participants:

• Children, elderly (retired) people, unemployed people, non-workers

• Restaurants outside of the worksite

• Participants with an allergy or a serious physical or mental illness

• Pregnant women

4. Excluded publications:

• Non-academic journals and reports

### Data extraction and management

Two authors (KS and SS) will independently screen the title and abstracts to find eligible studies. We will design a form to extract data. We will collect the full text of those eligible studies and two authors (KS and SS) will extract data independently to judge whether to include or exclude the studies. In the event of disagreement, KS and SS will consult with the other authors (EO, RM).

When unclear information regarding any of the data extraction is identified, we will attempt to contact the authors of the original reports to provide further details.

### Assessment of risk of bias in included studies

We will assess and report on risk of bias in accordance with the Cochrane Handbook for Systematic Reviews of Intervention [22] (Cochrane Handbook) for included studies. We will use the following criteria to assess the risk of bias: random sequence generation (to check for possible selection bias), allocation sequence concealment, outcome reporting, and other biases [22].

Further, KS and SS will evaluate independently whether or not to include studies in the meta-analysis. We will resolve disagreements through discussion and, if required, consult with a third author (EO). In the event of further disagreement, all authors (KS, SS, EO, and RM) will be consulted and a decision will be made based on consensus.

### Measures of treatment effect

#### ***Continuous data***

We will use the mean difference and 95% confidence intervals (CI) if outcomes are measured using the same technique between trials.

#### ***Dichotomous data***

For dichotomous data, we will present the results of the summary risk ratio and 95% CIs.

### Unit of analysis issues

#### ***Cluster-randomized trials***

For cluster RCTs, we will use the estimated value of the intracluster correlation co-efficient (ICC) obtained from experiments with similar features and adjust the sample size for each test in accordance with the Cochrane Handbook [22].

If we use ICCs from other sources, we will describe this and carry out sensitivity analysis in order to examine the random effect in the ICC. If we find both cluster RCTs and individual cluster RCTs, any relevant information will be incorporated. If we identify both cluster-randomized trials and individually randomized trials, we will conduct meta-analysis of the relevant information. In our review, we will include results from individual and cluster trials if minimal heterogeneity exists between the study designs and the interaction between the effects of intervention and if the choice of randomization unit is regarded as remote. In addition, we will explain the heterogeneity in the randomization unit and undertake a sensitivity analysis to explore the effects of the randomization unit.

### Dealing with missing data

For included studies, we will record levels of attrition. We will explore the impact of including studies with high levels of missing data in the primary outcome by using sensitivity analysis.

For all outcomes, intention-to-treat (ITT) analysis will be used as much as possible. All participants will be analyzed in the group to which they were allocated, regardless of whether or not they received the allocated intervention.

### Assessment of heterogeneity

We will evaluate heterogeneity in the meta-analyses using *I*^2^, *T*^2^, and *χ*^2^ statistics. We will consider that heterogeneity exists if *I*^2^ is 30% or more, *T*^2^ is greater than 0, or when the significance of *χ*^2^ is lower than 0.10.

### Assessment of reporting bias

If there are sufficient studies (10 or more) in the meta-analysis, we will investigate reporting biases (publication biases) using funnel plots. If asymmetry is identified or found in a visual assessment, we will verify asymmetry using exploratory analyses.

### Data synthesis

We will carry out statistical analysis using Review Manager V.5.3 (Cochrane Collaboration software). We will evaluate dichotomous data and continuous variables.

If the collected data from included studies shows statistical homogeneity, we will perform fixed-effect meta-analysis. If we anticipate the data to have significant heterogeneity between studies, we will perform random-effects meta-analyses if an average treatment effect across trials is considered clinically meaningful. We need to discuss the clinical implications of treatment effects if they differ between trials; and if the average treatment effect is not clinically meaningful, we will not synthesize trials further if we will use *I*^2^, *T*^2^, and *χ*^2^ statistics tests for assessing heterogeneity.

### Subgroup analysis and investigation of heterogeneity

We will investigate heterogeneity using subgroup analysis and sensitivity analysis.

We will implement subgroup analyses of the following:

1. Size of incentive: something that is taken away versus something that has to be paid out (e.g., “losing points” in increments if participants make unhealthy food choices versus making unhealthy food choices more expensive, such as a food tax).

2. Type of intervention:

• environmental intervention only versus environmental intervention including individual or group intervention.

• with calorie counts intervention versus without calorie counts intervention.

• incentive intervention versus other improvement intervention (e.g., free blood pressure checks or a company gymnasium).

3. Location of intervention:

• workplace cafeteria versus vending machines versus kiosk.

• urban versus suburban versus ex-urban versus rural.

4. Age: less than 29 years versus more than 30 years.

5. Sex: male versus female.

6. Size of the company/place of employment: a large global multinational company versus other company.

### Sensitivity analysis

As the potential risk of bias may affect the results and quality of studies included in the meta-analysis, we will perform a sensitivity analysis excluding the study’s two domains (concealment of allocation or incomplete outcome data) of risk of bias if the study is judged as a high risk of bias for primary outcomes.

For cluster RCTs, we will investigate the effect of random-effects or fixed-effects models using the ICC variable. In addition, we will conduct sensitivity analysis of the primary outcome only.

## Discussion

This review and meta-analysis will provide evidence of the effectiveness of targeted, population-level, food-based interventions in the workplace and will guide the future direction of interventions and research in nutrition education and health promotion.

## Competing interests

The authors declare that they have no competing interests.

## Authors’ contributions

KS and EO conceived of the review protocol and drafted the manuscript. SS and RM participated in the design of the review methods and commented on the draft. All authors read and approved the final manuscript.

## Supplementary Material

Additional file 1 **Search terms and strategies.** The search strategy utilized is outlined in more detail in the file. Click here for file

## References

[B1] Flegal KM Carroll MD Ogden CL Johnson CL Prevalence and trends in obesity among US adults 1999–2000 JAMA 2002 288 14 1723 1727 12365955 10.1001/jama.288.14.1723

[B2] Kelly T Yang W Chen C-S Reynolds K He J Global burden of obesity in 2005 and projections to 2030 Int J Obes (Lond) 2008 32 1431 1437 18607383 10.1038/ijo.2008.102

[B3] OECD Obesity rates among the adult population. Chapter on Health: Overweight and obesity OECD. OECD Fact Book 2014: Economic, Environmental and Social Statistics 2014 Paris: OECD Publishing 246 247 [http://dx.doi.org/10.1787/factbook-2014-102-en]

[B4] US Department of Health and Human Services (NCHS) Data Brief No. 131, October 2013: Prevalence of obesity among adults: United States*, 2011–2012* [http://www.cdc.gov/nchs/data/databriefs/db131.pdf] 24152742

[B5] Glanz K Mullis RM Environmental interventions to promote healthy eating: a review of models, programs, and evidence Health Educ Q 1998 15 395 415 3068206 10.1177/109019818801500403

[B6] World Health Organization (WHO) WHO Technical Manual on Tobacco Tax Administration 2010 Geneva: WHO [http://www.who.int/tobacco/publications/tax_administration/en/]

[B7] Jiménez-Ruiz JA Sáenz de Miera B Reynales-Shigematsu LM Waters HR Hernández-Ávila M The impact of taxation on tobacco consumption in Mexico Tob Control 2008 17 105 110 18285383 10.1136/tc.2007.021030

[B8] Dunlop SM Cotter TF Perez DA Impact of the 2010 tobacco tax increase in Australia on short-term smoking cessation: a continuous tracking survey Med J 2011 195 8 469 472 10.5694/mja11.10074 22004399

[B9] Briggs AD Mytton OT Madden D O’Shea D Rayner M Scarborough P The potential impact on obesity of a 10% tax on sugar-sweetened beverages in Ireland, an effect assessment modelling study BMC Public Health 2013 13 860 24044370 10.1186/1471-2458-13-860 PMC3852031

[B10] Briggs AD Mytton OT Kehlbacher A Tiffin R Rayner M Scarborough P Overall and income specific effect on prevalence of overweight and obesity of 20% sugar sweetened drink tax in UK: econometric and comparative risk assessment modelling study BMJ 2013 31 347:f6189 10.1136/bmj.f6189 PMC3814405 24179043

[B11] Mytton O Gray A Rayner M Rutter H Could targeted food taxes improve health? J Epidemiol Community Health 2007 61 8 689 694 17630367 10.1136/jech.2006.047746 PMC2652984

[B12] OECD Obesity update 2012 [http://www.oecd.org/health/49716427.pdf

[B13] Mayer JA Brown TP Heins JM Bishop DB A multi-component intervention for modifying food selections in a worksite cafeteria J Nutr Educ 1987 19 277 280

[B14] Zifferblatt SM Wilbur CS Pinsky JL A new direction for public health care: changing cafeteria eating habits J Am Diet Assoc 1980 76 15 20 7391441

[B15] Levin S Pilot study of a cafeteria program relying primarily on symbols to promote healthy choices J Nutr Educ 1996 28 282 285

[B16] Schmitz MF Fielding JE Point-of-choice nutritional labeling: evaluation in a worksite cafeteria J Nutr Educ 1986 18 S65 S68

[B17] French SA Jeffery RW Story M Breitlow KK Baxter JS Hannan P Snyder MP Pricing and promotion effects on low-fat vending snack purchases: the CHIPS Study Am J Public Health 2001 91 1 112 117 11189801 10.2105/ajph.91.1.112 PMC1446491

[B18] French SA Hannan PJ Harnack LJ Mitchell NR Toomey TL Gerlach A Pricing and availability intervention in vending machines at four bus garages J Occup Environ Med 2010 52 Suppl 1 S29 S33 20061884 10.1097/JOM.0b013e3181c5c476 PMC2818541

[B19] Lawrence S Boyle M Craypo L Samuels S The food and beverage vending environment in health care facilities participating in the Healthy Eating, Active Communities Program Pediatrics 2009 123 5 S287 S292 19470605 10.1542/peds.2008-2780G

[B20] Lowe MR Tappe KA Butryn ML Annunziato RA Coletta MC Ochner CN Rolls BJ An intervention study targeting energy and nutrient intake in worksite cafeterias Eat Behav 2010 11 3 144 151 20434060 10.1016/j.eatbeh.2010.01.002 PMC2901864

[B21] Moher D Liberati A Tetzlaff J Altman DG PRISMA Group Preferred reporting items for systematic reviews and meta-analyses: the PRISMA statement Ann Intern Med 2009 151 4 264 269 19622511 10.7326/0003-4819-151-4-200908180-00135

[B22] Higgins JPT Green S Cochrane Handbook for Systematic Review of Interventions, 5.1.0 2011 Chichester: The Cochrane Collaboration

