# Supplementary material for: Financial incentive policies at workplace cafeterias for preventing obesity—a systematic review and meta-analysis (Protocol)
Source: Syst Rev. 2014 Oct 28;3:128. doi: 10.1186/2046-4053-3-128 (PMC4274765; doi:10.1186/2046-4053-3-128)
Supplement: Additional file 1 — Search terms and strategies. The search strategy utilized is outlined in more detail in the file. [file 2046-4053-3-128-S1.doc]

**Additional file 1: Search terms and strategies**

| **Database Field Guide Ovid MEDLINE(R) In-Process & Other Non-Indexed Citations July 23, 2014,**  **Database Field Guide Ovid MEDLINE(R) 1946 to Present with Daily Update,**  **Database Field Guide Ovid OLDMEDLINE(R) 1946 to 1965**  **1. Workplace/**  **2. Work/**  **3. work*.tw.**  **4. Occupations/**  **5. occupat*.tw.**  **6. job*.tw.**  **7. Employment/**  **8. employ*.tw.**  **9. Industry/**  **10. industr*.tw.**  **11. Organizations/**  **12. organization*.tw.**  **13. middle aged/**  **14. middle age*.tw.**  **15. young adult/**  **16. young adult*.tw.**  **17. office*.tw.**  **18. busines*.tw.**  **19. (company or companies).tw.**  **20. human resourc*.tw.**  **21. adult*.tw.**  **22. staff*.tw.**  **23. colleague*.tw.**  **24. enterpri?e*.tw.**  **25. (factory or factories).tw.**  **26. or/1-25**  **27. Food Dispensers, Automatic/**  **28. food dispenser*.tw.**  **29. cafe*.tw.**  **30. kiosk*.tw.**  **31. vending machine*.tw.**  **32. vending snack*.tw.**  **33. canteen*.tw.**  **34. buffet*.tw.**  **35. (deli or delis).tw.**  **36. (milk bar or milk bars).tw.**  **37. Meals/**  **38. lunch*.tw.**  **39. Restaurants/**  **40. restaurant*.tw.**  **41. (store or stores).tw.**  **42. shop*.tw.**  **43. or/27-42**  **44. Environment/**  **45. environment*.tw.**  **46. Motivation/**  **47. motivation*.tw.**  **48. incentive*.tw.**  **49. exp Health Promotion/**  **50. (promotion* adj2 item*).tw.**  **51. (wellness* adj2 program*).tw.**  **52. (health* adj2 campaign*).tw.**  **53. (promotion* adj2 health*).tw.**  **54. exp Reward/**  **55. reward*.tw.**  **56. bonus point*.tw.**  **57. discount*.tw.**  **58. sale*.tw.**  **59. Social Marketing/**  **60. social market*.tw.**  **61. "Marketing of Health Services"/**  **62. market*.tw.**  **63. coupon*.tw.**  **64. voucher*.tw.**  **65. price*.tw.**  **66. cash back*.tw.**  **67. benefit*.tw.**  **68. Behavioral Sciences/**  **69. (behavior* adj2 science*).tw.**  **70. or/44-69**  **71. randomized controlled trial.pt.**  **72. controlled clinical trial.pt.**  **73. randomized.ab.**  **74. placebo.ab.**  **75. drug therapy.fs.**  **76. randomly.ab.**  **77. trial.ab.**  **78. groups.ab.**  **79. or/71-78**  **80. 26 and 43**  **81. 70 and 80**  **82. 79 and 81**  **83. exp animals/ not humans.sh.**  **84. 82 not 83**  **85. remove duplicates from 84** |
| --- |
